# The Absence of Sensory Axon Bifurcation Affects Nociception and Termination Fields of Afferents in the Spinal Cord

**DOI:** 10.3389/fnmol.2018.00019

**Published:** 2018-02-08

**Authors:** Philip Tröster, Julia Haseleu, Jonas Petersen, Oliver Drees, Achim Schmidtko, Frederick Schwaller, Gary R. Lewin, Gohar Ter-Avetisyan, York Winter, Stefanie Peters, Susanne Feil, Robert Feil, Fritz G. Rathjen, Hannes Schmidt

**Affiliations:** ^1^Developmental Neurobiology, Max Delbrück Center for Molecular Medicine in the Helmholtz Association, Berlin, Germany; ^2^Molecular Physiology of Somatic Sensation, Max Delbrück Center for Molecular Medicine in the Helmholtz Association, Berlin, Germany; ^3^Institute of Pharmacology, College of Pharmacy, Goethe University, Frankfurt am Main, Germany; ^4^Institute of Pharmacology and Toxicology, Zentrum für Biomedizinische Ausbildung und Forschung (ZBAF), Witten/Herdecke University, Witten, Germany; ^5^Cognitive Neurobiology, Humboldt University of Berlin, Berlin, Germany; ^6^Interfaculty Institute of Biochemistry, University of Tübingen, Tübingen, Germany

**Keywords:** sensory neurons, axon bifurcation, Npr2, cGKI, development, nociception and pain, axonal pathfinding

## Abstract

A cGMP signaling cascade composed of C-type natriuretic peptide, the guanylyl cyclase receptor Npr2 and cGMP-dependent protein kinase I (cGKI) controls the bifurcation of sensory axons upon entering the spinal cord during embryonic development. However, the impact of axon bifurcation on sensory processing in adulthood remains poorly understood. To investigate the functional consequences of impaired axon bifurcation during adult stages we generated conditional mouse mutants of Npr2 and cGKI (*Npr2*^*fl*/*fl*^*;Wnt1*^*Cre*^ and *cGKI*^*KO*/*fl*^*;Wnt1*^*Cre*^) that lack sensory axon bifurcation in the absence of additional phenotypes observed in the global knockout mice. Cholera toxin labeling in digits of the hind paw demonstrated an altered shape of sensory neuron termination fields in the spinal cord of conditional Npr2 mouse mutants. Behavioral testing of both sexes indicated that noxious heat sensation and nociception induced by chemical irritants are impaired in the mutants, whereas responses to cold sensation, mechanical stimulation, and motor coordination are not affected. Recordings from C-fiber nociceptors in the hind limb skin showed that Npr2 function was not required to maintain normal heat sensitivity of peripheral nociceptors. Thus, the altered behavioral responses to noxious heat found in *Npr2*^*fl*/*fl*^*;Wnt1*^*Cre*^ mice is not due to an impaired C-fiber function. Overall, these data point to a critical role of axonal bifurcation for the processing of pain induced by heat or chemical stimuli.

## Introduction

The primary sensory representation of the body within the central nervous system is based on the intricate innervation patterns of dorsal root ganglion (DRG) neurons into the spinal cord. This projection represents an attractive system to study the branching of axons and has enabled the characterization of a cGMP-dependent signaling cascade essential for the bifurcation of sensory axons. This specific form of neuronal branching proceeds during embryonic development at the so-called dorsal root entry zone (DREZ) where axons of DRG neurons split into ascending and descending stem axons that grow along the lateral margin of the spinal cord (Brown, 1981). Previous investigations showed that the cGMP signaling cascade composed of C-type natriuretic peptide (CNP), the receptor guanylyl cyclase Npr2 (also designated GC-B or Npr-B), and the cGMP-dependent protein kinase I (cGKI, also known as PKGI) are essential for the bifurcation of axons from DRG as well as cranial sensory ganglion neurons (Schmidt et al., 2002, 2007, 2009; Zhao and Ma, 2009; Zhao et al., 2009; Ter-Avetisyan et al., 2014). In the absence of any one of these components sensory axons no longer bifurcate and instead turn either in a rostral or caudal direction. Consistent with these observations is the timing and pattern of localization of CNP in the dorsal spinal cord and Npr2 and cGKI in sensory neurons (Schmidt et al., 2009). A critical missing link of the Npr2-mediated cGMP signaling pathway is the characterization of phosphorylation targets of cGKIα in sensory growth cones that mediate axon bifurcation. Such data might provide mechanistic insights into the machinery for bifurcation. The nitric oxide-sensitive guanylyl cyclases (NO-GCs) are not expressed in embryonic DRG neurons and thus not implicated in sensory axon branching *in vivo* (Schmidt et al., 2007, 2009). Also, variations in cGMP levels caused by the absence of phosphodiesterase 2A do not interfere with proper bifurcation of sensory axons (Schmidt et al., 2016). Notably, collateral sprouting originating from the stem axons as well as branch formation in the periphery of the body are not affected by the absence of Npr2-mediated cGMP signaling (Schmidt et al., 2007, 2009; Ter-Avetisyan et al., 2014).

Additionally, CNP and Npr2 are also involved in the process of endochondral ossification which is essential for long bone growth. Consequently, biallelic loss-of-function mutations including missense, nonsense, frame-shift mutations, insertions and deletions, and splice site mutations in the human *Npr2* gene result in acromesomelic dysplasia type Maroteaux (AMDM; OMIM602875), a skeletal dysplasia with an extremely short and disproportionate stature (Bartels et al., 2004; Potter, 2011; Kuhn, 2016). Whether AMDM patients also reveal bifurcation errors of sensory axons when entering the spinal cord is currently not known and unfortunately, neurological qualities have so far not been characterized on patients with mutations in the *Npr2* gene. Similarly to human patients, constitutive Npr2-deficient mice show dwarfism (Chusho et al., 2001; Tamura et al., 2004; Tsuji and Kunieda, 2005). Due to their decreased survival rate at post-weaning stages, *Npr2* global mouse knockouts are of limited use for further anatomical, physiological, and behavioral studies. Furthermore, the disproportionate bone growth in Npr2-deficient mice might cause the vertebrate column to squeeze on spinal or cranial nerves which in turn might affect sensation. Unlike Npr2 and its ligand CNP, cGKI is not implicated in long bone growth. However, constitutive cGKI-deficient mice have a number of other deficits including gastrointestinal and cardiovascular impairments which limits their use for investigations on the impact of disturbed axon bifurcation on sensory information processing (Hofmann et al., 2006).

To study the functional consequences of the absence of axon bifurcation in the spinal cord in the absence of other phenotypes that may complicate the interpretation of results, we have generated floxed alleles of *Npr2* and *cGKI* for conditional inactivation of *Npr2* or *cGKI* in DRG neurons at early stages. As in constitutive *Npr2* or *cGKI* knockout mice, DRG axons from mice with a conditional inactivation of *Npr2* or *cGKI* in the neural crest completely lack bifurcations which was associated with altered spinal termination patterns. Behavioral testing indicated that noxious heat sensation as well as nociception induced by chemical irritants was impaired, whereas the behavior driven by mechanical stimulation or balance of body position and motor coordination remained unchanged in mice without sensory axon bifurcations.

## Materials and methods

### Mice

#### Generation of *Npr2^*fl*/*fl*^* mice

**Figure 1:**
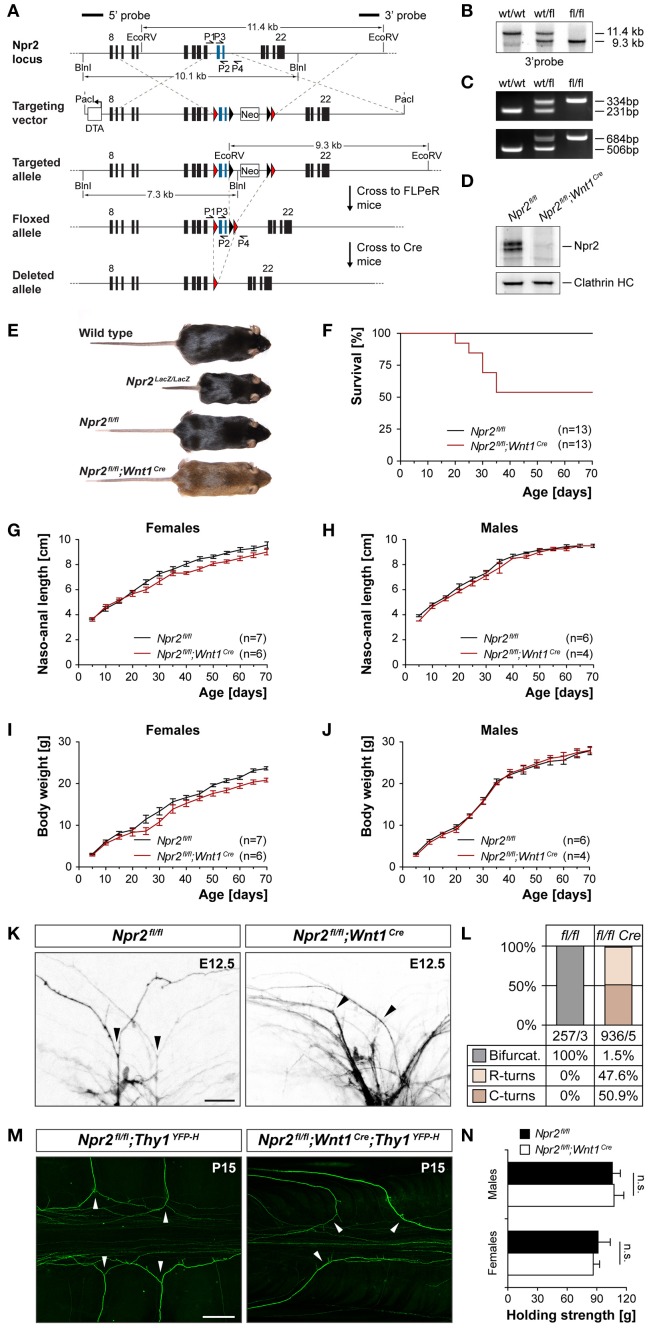
Generation of a conditional *Npr2*-deficient mouse mutant and analysis of sensory axon bifurcation at the DREZ. **(A)** Targeting strategy for the generation of the floxed *Npr2* allele and inactivation by Wnt1-Cre. **(B)** Southern blotting of the wild type, heterozygotes, and homozygous floxed allele using genomic DNA isolated from liver tissue. **(C)** PCR genotyping of wild type, heterozygotes and homozygous allele. Upper panel primer P1 and P2 flanking the loxP1 site, lower panel primer P3 and P4 flanking loxP2. **(D)** Western blotting of membrane fractions from E13.5 DRGs demonstrating the absence of Npr2 protein after recombination with Wnt1-Cre. The heavy chain of clathrin served as loading control. **(E)** Depiction of wild type, *Npr2*^*LacZ*/*lacZ*^, *Npr2*^*fl*/*fl*^ or *Npr*^2*fl*/*fl*^*;Wnt1*^*Cre*^ mice of mature stages (see also Figure S1). **(F)** Survival rate of *Npr2*^*fl*/*fl*^ or *Npr2*^*fl*/*fl*^*;Wnt1*^*Cre*^ mice at different ages. Numbers of animals inspected are given in parenthesis. **(G–J)** Naso-anal length and body weight of *Npr2*^*fl*/*fl*^ or *Npr2*^*fl*/*fl*^*;Wnt1*^*Cre*^ males and females at different ages. **(K)** Analysis of DRG axon bifurcation by DiI tracing in *Npr2*^*fl*/*fl*^ or *Npr2*^*fl*/*fl*^*;Wnt1*^*Cre*^ E12.5 embryos (see also Figure S2). Arrow heads mark bifurcations or turns of sensory axons in wild type or mutants, respectively. Bar, 25 μm. **(L)** Quantification of bifurcation errors demonstrated the complete absence of DRG axon bifurcation in *Npr2*^*fl*/*fl*^*;Wnt1*^*Cre*^ E12.5 embryos. The numbers of counted axons and embryos are indicated below the columns. **(M)** Bifurcation errors persisted at mature stages (P15) as indicated by the reporter *Thy1-YFP-H* in the *Npr2*^*fl*/*fl*^*;Wnt1*^*Cre*^*;Thy1*^*YFP*−*H*^ mouse. Arrow heads mark bifurcations or turns of sensory axons in wild type or mutants, respectively. Bar, 250 μm. **(N)** Analysis of grip strength revealed indistinguishable maximal muscle strength (mean ± SEM) of the forelimbs between mature *Npr2*^*fl*/*fl*^ (*n* = 6 males and 6 females) and *Npr2*^*fl*/*fl*^*;Wnt1*^*Cre*^ (*n* = 8 males and 7 females) of both sexes (paired *t*-test; *p* = 0.90 and 0.72 in males and females, respectively).

Based on the bacterial artificial chromosome clone bMQ331a20 (BioScience) two loxP sites were inserted by standard procedures into the intronic sequences flanking exons 17 and 18 of the murine *Npr2* gene to generate the target construct. R1 embryonic stem cells (ESCs) (129X1x129S1) were electroporated and clones that had incorporated the targeting vector into their genome were selected by G418 and analyzed for homologous recombination after digestion with EcoRV or BlnI by Southern blotting using either the 3′ or 5′ probe as indicated in Figure 1A. ESCs were injected into blastocysts from C57Bl/6 mice and chimera that transmitted the floxed *Npr2* allele were identified by Southern blotting and by PCR genotyping using the following primer sequences: P1 5-GCCACTTTTGCACCCGGATG-3, P2 5-GTGACGCTGTCGAAGGCCTC-3, P3 5-CCTGCTTTGATGCCATTATCG-3, and P4 5-CTGCAACAACCAAAGCTCAG-3. Crossbreeding with Flpe-deleter mice induced the excision of the neomycin cassette flanked by FRT sites (B6; 129S7-*Npr2*^*tm*4(*flox*)*Fgr*^).

Genotyping of the floxed cGKI mouse (B6.129-*Prkg1*^*tm*2*Naw*^) (Wegener et al., 2002) (RRID:MGI:2668654), *Thy1-YFP-H* (*Tg(Thy1-YFP)H*^*Jrs*/*J*^) (Feng et al., 2000) (RRID:IMSR_JAX:003782), *Wnt1-Cre (Tg(Wnt1-Cre)11Rth)* (Danielian et al., 1998) (RRID:IMSR_JAX:003829), FLPE-deleter strain (B6.129S4-*Gt(ROSA)26Sor*^*tm*1(*FLP*1)*Dym*^/RainJ; The Jackson Laboratories) (Farley et al., 2000) (RRID:IMSR_JAX:009086), *TrkA-Cre* mice (B6;129S4-*Ntrk1*^*tm*1(*cre*)*Lfr*^/Mmucd; Mutant Mouse Regional Resource Centers, https://www.mmrrc.org/) (Quina et al., 2015) (RRID:MMRRC_015500-UCD), *TrkC-Cre* mice (B6.129X1-*Ntrk3*^*tm*1(*cre*)*Lfr*^*/*Mmucd, originating in the laboratory of Louis Reichardt, provided by Karina Gültig, University of Tübingen) (Funfschilling et al., 2004) (RRID:MMRRC_000364-UCD), *Npr2-LacZ* (B6.129P2-*Npr2*^*tm*1.1(*nlslacZ*)/*Fgr*^) (Ter-Avetisyan et al., 2014) (RRID:MGI:5568090), *Npr2-CreERT2* (B6.129S7-*Npr2*^*tm*1.2(*CreERT*2)/*Fgr*^) (Ter-Avetisyan et al., 2014) or *Rosa26-tdTomato* (B6.Cg-*Gt(ROSA)26Sor*^*tm*14(*CAG*−*TdTomato*)*Hze*^/J) (Madisen et al., 2010) (RRID:IMSR_JAX:007914) was performed by PCR as described.

Animals were housed on a 12/12 h light/dark cycle with free access to food. The animal procedures were performed according to the guidelines from directive 2010/63/EU of the European Parliament on the protection of animals used for scientific purposes. All experiments were approved by the local authorities of Berlin(LaGeSO) (numbers T0313/97, 0143/07, G0370/13, G0239/11, X9014/15, and G0222/14), the Landesamt für Natur, Umwelt und Verbraucherschutz Nordrhein-Westfahlen (number 84-02.04.2012.A422), and Regierungspräsidium Darmstadt (number FU/1102). All experiments were also approved by the local Ethics Committee for Animal Research and adhered to the guidelines of the Committee for Research and Ethical Issues of the International Association for the Study of Pain.

### Axon tracing by DiI, immunohistochemistry, and western blotting

DiI tracing and immunofluorescent staining of cryostat sections (16 μm) from paraformaldehyde-fixed tissue were performed as described previously (Schmidt et al., 2007; Schmidt and Rathjen, 2011).

The following primary and secondary antibodies using the indicated concentrations or dilutions were applied to tissue sections or Western blotting: Guinea pig antiserum to the extracellular domain of Npr2 (dilution: 1:5,000) (Ter-Avetisyan et al., 2014), guinea pig antiserum to amino acids 2-89 of cGKIα (1:25,000 for immunofluorescence staining), affinity-purified rabbit antibody to amino acids 2-89 of cGKIα (0.25 μg/ml for Western blot analysis), rabbit antibody to full length cGKIα expressed in Sf9 cells (1 μg/ml of the IgG fraction for Western blot analysis), chicken anti-β-galactosidase (1:5,000; Abcam, ab 9361; RRID:AB_307210), mouse anti-clathrin heavy chain (0.05 μg/ml; BD Biosciences, 610499; RRID:AB_397865), mouse anti-GAPDH (1:7,500; Novus Biologicals, NB300-221; RRID:AB_10077627), rabbit anti-PGP9.5 (1:1,000, Dako, Z5116, RRID:AB_2622233), rabbit anti-trkA (1:1,000; Millipore, 06-574; RRID:AB_310180), rabbit anti-parvalbumin (1:1,000; Swant, PV28; RRID:AB_2315235), rabbit anti-CGRP (1:1,000; Chemicon, AB1971; RRID:AB_2313629), rabbit anti-red fluorescent protein (1:2,500, ABIN129578; RRID:AB_10781500), isolectin GS-IB4-Alexa-488 (1:50; Life Technologies; RRID:AB_2314662), donkey anti-chick-IgY-Cy3 (1:1,000; Dianova), goat-anti-rabbit-Alexa488 (1:1,000; Dianova), goat anti-guinea pig-Alexa488 (1:1,000; Dianova), donkey anti-guinea pig-HRP (Dianova), goat anti-rabbit-HRP, and goat anti-mouse-HRP (all 1:20,000; Dianova,). 0.1 mg per g body weight of tamoxifen was applied by oral gavaging as described (Ter-Avetisyan et al., 2014).

Microscopic images were obtained at room temperature by confocal imaging using a Carl Zeiss LSM 710 NLO Laser Scanning Microscope equipped with ZEN 2010 software and the following lenses: a Plan-Neofluar 10x/0.30 NA objective, a Plan-Achromat 40x/1.40 NA oil objective, or a Plan-Achromat 63x/1.40 NA oil objective (all from Carl Zeiss MicroImaging, GmbH). Images were imported into Photoshop CS5 (Adobe) for uniform adjustment of contrast and brightness. Figures were assembled using Illustrator CS5 (Adobe).

### Transganglionic labeling using cholera toxin subunit B, optical clearing, and imaging of fixed spinal cord tissue

Five to six-week old mice of either sex were anesthetized by an intraperitoneal injection of ketamine (100 mg/kg) and xylazine (10 mg/kg). 0.2 μl of 1.5% cholera toxin subunit B conjugated with Alexa Fluor 594 (CTB-AL594) (Wan et al., 1982; Robertson and Arvidsson, 1985; Conte et al., 2009) in 0.1 M phosphate buffered saline (PBS) were injected subcutaneously into the plantar surface of the left second hindpaw digit using a pulled glass capillary attached to a Hamilton microliter syringe. The glass capillary was inserted into the most distal interphalangeal crease and advanced under the skin toward the next proximal crease where the tracer was slowly injected. Five days post-injection, allowing for transganglionic transport of the tracer, mice were transcardially perfused with PBS and ice-cold 4% paraformaldehyde (PFA). Subsequently, the lumbar spinal cord was dissected out and post-fixed overnight in 4% PFA at 4°C. Fixed spinal cords were washed three times with 0.1 M PBS for 10 min each at RT. Subsequently, the tissue was immersed in ascending concentration series of 2,2'-thiodiethanol (TDE) (Staudt et al., 2007; Kloepper et al., 2010; Aoyagi et al., 2015; Costantini et al., 2015) for 24 h each at RT. The applied concentrations were 10, 25, 50, and 97% TDE diluted with 0.1 M PBS. During all incubation steps, the samples were kept on a vibrating table in the dark. For two-photon imaging, the cleared spinal cords were mounted on glass slides in 97% TDE which has a refractive index of 1.52 (Staudt et al., 2007) using press-to-seal silicone isolators with the dorsal surface facing up.

Two-photon imaging was performed using a laser scanning microscope equipped with a tunable Ti:sapphire laser. Two channels were recorded sequentially to collect Alexa Fluor 594 fluorescence (excitation wavelength: 810 nm; emission range: 600–735 nm) and tissue autofluorescence (excitation wavelength: 810 nm; emission range: 505–575 nm). A 25x multi-immersion objective (0.8 numerical aperture) was used with immersion oil for cleared sample imaging. Tiled stacks were taken through the spinal cord dorsal horn (pixel size: 0.5 × 0.5 μm; z step size: 1.5 μm). All images were processed using ImageJ (Schneider et al., 2012). Tiled stacks were stitched using either the imaging software ZEN 2010 or the ImageJ plugin “Stitching 2D/3D” (Preibisch et al., 2009). Subsequently, images were cropped to the same size and reduced to the same slice number. Next, background fluorescence was reduced by subtracting the autofluorescence channel from the CTB-AL594 channel. Using stack histogram-based thresholding, the image stacks were binarized. The threshold was set as the mean gray value plus three times the standard deviation. Finally, single pixels were removed to reduce noise, e.g., hot pixels. In order to enable comparative analyses of spinal terminal fields of cutaneous myelinated afferents, the three-dimensional centers of mass of the voxel clouds representing CTB-AL594 labeled fiber terminals were determined using the ImageJ plugin “3D ImageJ Suite” (Ollion et al., 2013). All images were aligned to the center of mass of the voxel cloud, i.e., images were cropped to the same size and reduced to the same slice number around the respective centers of mass. Summed dorsoventral, rostrocaudal, and/or mediolateral projections of the binary image stacks were constructed to enable two-dimensional visualization of terminal fields.

#### Morphometric analysis of spinal terminal fields

Mediolateral, rostrocaudal, and dorsoventral spans of the terminal fields were measured in summed dorsoventral and rostrocaudal projections of binary image stacks. Summed projections were thresholded with the threshold being set as the mean gray value plus one standard deviation. Subsequently, the dimensions of the bounding rectangles enclosing all pixels representing CTB-AL594 labeled fiber terminals were measured.

#### Density analysis of spinal terminal fields

Areal densities (as voxels per area) of spinal terminal fields were calculated in summed dorsoventral, mediolateral, and rostrocaudal projections of binary image stacks. Using the ImageJ plugin “3D ImageJ Suite” (Ollion et al., 2013), the total number of voxels representing CTB-AL594 labeled fiber terminals was determined in binary image stacks. Subsequently, the number of voxels was divided by the area (in μm^2^) that was occupied by positive pixels in summed dorsoventral, mediolateral, and rostrocaudal projections, respectively.

### *Ex vivo* skin nerve preparation

*Ex vivo* skin nerve preparations were performed as described (Moshourab et al., 2013). Briefly, mice were sacrificed and the glabrous skin on the left hindlimb was removed. The saphenous nerve was exposed dissected free along the lower leg up. Subsequently, the skin was carefully removed from the musculoskeletal and the connective tissue of the paw. The skin-nerve preparation was placed in an organ bath filled with oxygenated 32°C warm synthetic interstitial fluid (SIF) consisting of 123 mM NaCl, 3.5 mM KCl, 0.7 mM MgSO_4_, 1.7 mM NaH_2_PO_4_, 2.0 mM CaCl_2_, 9.5 mM sodium gluconate, 5.5 mM glucose, 7.5 mM sucrose and 10 mM HEPES, at a pH of 7.4. Using insect needles, the skin was mounted in the organ bath with its epidermis facing the bottom of the chamber, exposing the dermis to the solution. The nerve was pulled through a hole into the adjacent recording chamber which was filled with mineral oil. Finally, using fine forceps the nerve was desheathed by removing its epineurium and small filaments were teased of the nerve. Throughout the whole experiment the skin was superfused with oxygenated SIF at a flow rate of 15 ml/min.

#### Single-unit recordings

Single receptor units were recorded as previously described (Moshourab et al., 2013). Teased filaments were attached to a recording electrode and the receptive fields (RF) of individual units were identified by manually probing the skin with blunt forceps. To probe for heat-responsive units, 1 ml of hot (48°C) SIF buffer was washed over the surface of the skin. For immediate visual identification of single units, whole action potential waveforms were resolved on an oscilloscope. Data was acquired using a PowerLab 4/30 system which was controlled with the software LabChart 7.1.The conduction velocities (CVs) of single fibers were determined by evoking a local action potential with a platinum iridium electrode. The electrical impulse was conducted nearly instantaneously through the solution whereas the triggered action potential conducted by the fiber was delayed depending on the fiber type and the distance of the fiber's receptive field from the electrode. Hence, the distance between the RF of a unit to the electrode was measured and the CV was calculated as distance divided by time delay. Fibers with CVs below 1.3 m/s were classified as C-fibers.

#### Stimulation protocols

A Peltier device (custom device built by the Yale School of medicine Instrument Repair and Design) was used to apply warm or cool stimulation parameters to the RFs of cutaneous afferent fibers. Two heat ramps were applied to skin: firstly a ramp and hold stimulus from 32 to 48°C with a 0.5 s ramp, followed by a 3 s hold time and a further 0.5 s ramp; and secondly a slow 15 s ramp from 32 to 48°C. Two cold ramps were also applied: first a ramp and hold stimulus from 32 to 12°C with a 0.5 s ramp, followed by a 3 s hold time, and a further 0.5 s ramp; and secondly a slow 15 s ramp from 32 to 12°C.

A computer-controlled Nanomotor (Kleindieck, Germany) was used to apply controlled mechanical stimuli of known amplitude and velocity to the RF of afferent fibers. Four 3 s ramp and hold stimuli were applied to the RF of each afferent fiber, each increasing in amplitude (40, 75, 150, 220 mN).

#### Analysis of extracellular afferent recordings

Thermosensitive C-fibers were searched for in recordings and for further analysis. Spike sorting was performed with a spike analysis plug-in of the LabChart software (AD Instruments). C-mechanoheat (C-MH), C-mechanoheatcold (C-MHC), and C-mechanocold (C-MC) fibers were pooled in each experimental group (Milenkovic et al., 2014). Thermal thresholds represent the temperature required to cause the first action potential spike in slow (15 s) heat and cold ramp protocols. Mechanical thresholds represent the average force required to cause the first action potential spike over the four ramp and hold stimuli. Mean group data were compared using *t*-tests and two-way repeated measures ANOVA tests.

### Behavioral testing

Littermate mice of either sex were used in all behavioral tests (8 to 10-weeks old). The *Npr2*^*fl*/*fl*^*;Wnt1*^*Cre*^ mice were on a mixed genetic background (Bl6/SV129) and *cGKI*^*KO*/*fl*^*;Wnt1*^*Cre*^ mice were on a Bl6 background. Animals were habituated to the experimental room and were investigated by observers blinded for the genotype.

#### Hot plate test

Mice were placed into a Plexiglas cylinder on a metal surface maintained at 50, 52, or 54°C (Hot Plate; Ugo Basile, Comerio Italy). Cut-off times were 60, 40, and 20 s, respectively, to prevent tissue damage. The time between placement and shaking or licking of the hindpaws or jumping off the plate was recorded.

#### Acetone test

For acetone-evoked evaporative cooling, animals were habituated in a glass enclosure on a mesh floor, a drop of acetone (50 μl) was applied to the plantar side of a hindpaw and responses were observed for 1 min. The responses were scored as follows: 0, no response (sniffing or walking was not considered nociceptive); 0.5, a licking response; 1, flinching and brushing of the paw; 2, strong flinching (Caspani et al., 2009).

#### Capsaicin test

Capsaicin (5 μg dissolved in 10 μl of a solution consisting of 90% sterile saline, 5% ethanol, and 5% Tween 80) was injected subcutaneously (s.c.) into the dorsal surface of a hindpaw. The time spent licking the capsaicin-injected paw and the number of shakes of the injected paw was recorded for 5 min.

#### Formalin test

Formalin (20 μl of a 0.5% formaldehyde solution) was s.c. injected into the dorsal surface of a hindpaw (Braz and Basbaum, 2010). The time spent licking the formalin-injected paw was recorded in 5 min intervals up to 60 min after formalin injection.

#### Hargreaves test

The Hargreaves test was performed with an idle value of 5% and a stimulus value of 50% of the maximum intensity as described (Hargreaves et al., 1988). The withdrawal reaction induced by heat stimulation was measured 25 times per animal over period of 3 consecutive days using model 400 Heated Base (IITC Life Science Inc.).

#### Dynamic plantar test

The mechanical sensitivity of the plantar side of a hindpaw was assessed with an automated von Frey-type testing device (Dynamic Plantar Aesthesiometer; Ugo Basile). This device pushes a thin probe (0.5 mm diameter) with increasing force through a wire-grated floor against the plantar surface of the paw from beneath, and it automatically stops and records the latency time, after which the animal withdraws the paw. The force increased from 0 to 5 g within 10 s (0.5 g/s ramp) and was then held at 5 g for an additional 10 s (Lu et al., 2015). The paw withdrawal latency was calculated as the mean of five to six consecutive trials with at least 20 s in-between.

### Balance and motor coordination in the absence of Npr2

#### Grip strength assay

Motor functions were assessed using the grip strength assay that measures maximal muscle strength of the forelimbs (TSE Grip Strength Meter). An animal holding onto the meter handle was pulled backwards steadily in horizontal orientation, and the mean of three maximum force measurements was recorded.

Balance and coordination were examined using the beam walking test, rotarod performance test, staircase test, adhesion removal test, food grasping and reaching test, and walking track (MouseWalker) which provide readouts for general motor performance.

#### The beam walking test

The beam walking test assesses motor coordination and balance from the ability of mice to traverse a series of narrow, horizontal beams graded in diameter. Wooden beams were 1 m × 10 or 15 or 20 mm diameter at 50 cm height with one starting end (with a 60 W aversive light) and the other end attached to an escape box. Mice were trained for 2 days with four trials per day, motivated by a reward in the escape box until traversing took < 20 s. On the test day (day 3) mice received two consecutive trials per beam, progressing from wide to narrow, allowing up to 60 s for a traversal. A fall off counted as incomplete trial. Durations of beam traversals und instances of hind feet slipping off the beam were recorded (Carter et al., 2001; Luong et al., 2011).

#### The staircase test

The staircase test (Campden Instruments) allows measurement of lateralized effects on skilled motor function required for reaching and grasping a food pellet from variable distance. Food was removed from subjects' home cages 4–6 h before first training and prior to later experiments animals were fed one pellet of regular feed. Animals were habituated to sucrose pellet rewards at least three times prior to testing to avoid food neophobia. Mice were trained for 3 days in the staircase test box with the double, eight-step staircase baited with two pellets per step (16 on each side, total 32). Testing was conducted with one pellet per step and sessions lasted for 15 min. We recorded collected pellets, and maximum distance reached.

#### The adhesion removal test

The adhesion removal test assesses sensory and motor deficits related to paw and mouth coordination. It was performed in an empty mouse cage where a mouse received two adhesive tape strips (3 × 4 mm) onto the hairless part of the forepaws. The time to first contact reaction was recorded for both paws, as well as the time to tape removal.

#### The reaching and grasping test

The reaching and grasping test was performed with an automated forepaw reaching chamber (Campden Instruments) providing sensitive measures of fine motor coordination (Marques and Olsson, 2010). Prior to testing subjects were habituated to sucrose pellets. Four days before testing, animals were trained daily to reach for pellets, each training lasting until either 10 pellets were retrieved or 10 min had passed. During the test one pellet at a time was placed in the tray. Reaching attempts were counted until the pellet was removed. Performance parameters were latency to first reach, reaching accuracy (pellets retrieved per reach attempt), latency to first pellet retrieval, interval to retrieve five pellets after the first pellet had been retrieved.

#### The rotarod test

The Rotarod test was performed using Rota-Rod 47600 (Ugo Basile) with an acceleration of the rotating cylinder from 5 to 40 rpm in 360 s. Animals were given 2 days of habituation to the Rotarod (same protocol as in test). Four recordings per day were done.

The mouse's gait was tested using a home-made *MouseWalker* as described in Mendes et al. (2015). This system is based on the total internal reflection of light on transparent plates. Paw contacts of the mice disrupt this reflection and generate scattered light which is then detected by a high speed digital camera. Parameters such as foot print clustering and stance linearity were evaluated.

### Experimental design and statistical analysis

Sample size for behavioral experiments and for analysis of axon branching was deduced from previously published studies (Schmidt et al., 2007) or in publications listed above in the sub-section on behavioral testing. No further statistical methods were used to predetermine sample size. Sample size are given directly in the figures or in the legends. Experiments were done blind with respect to genotype. Littermates were used as control. Statistical analysis was performed with SPSS software (RRID:SCR_002865) using the Student's *t*-test for paired comparisons or Mann-Whitney *U*-test. Power analysis of statistical data was performed with the software “nQuery + nTerim 4.0.” Depending on the equality or inequality of variances and on the equality and inequality of the number of measurements *t*-test or Satterthwaite *t*-test were applied.

## Results

### Conditional inactivation of *Npr2* in DRG neurons interrupted sensory axon bifurcation

To generate a conditional allele of *Npr2*, two loxP sites were introduced into the introns flanking exons 17 and 18 of the mouse *Npr2* gene which encode the guanylyl cyclase domain of Npr2. The neo selection cassette flanked by two FRT sites was removed by crossing this mouse line with a FLPE-deleter strain. The resulting floxed *Npr2* allele contains two loxP sites surrounding exon 17 and 18 (Figure 1A). The correct integration of the loxP sites was verified by Southern blotting using (Figure 1B) a probe as indicated in Figure 1A and PCR was applied for genotyping using two distinct primer pairs that amplify genomic regions including loxP1 or loxP2, respectively (Figure 1C). Conditional inactivation of *Npr2* in DRG neurons at early embryonic stages when sensory axons enter the spinal cord was obtained by crossbreeding with the *Wnt1-Cre* transgenic mouse line. At early embryonic stages Wnt1 is restricted to the midbrain, dorsal spinal cord and DRGs as detected by the *Wnt1-lacZ* reporter mouse (Echelard et al., 1994). Cre-mediated excision of the exon 17 and exon 18 generated a frameshift in exon 19 which leads to a premature stop codon. This resulted in nearly undetectable expression of Npr2 protein in DRGs as indicated by Western blotting using antibodies to the extracellular domain of Npr2 (Figure 1D).

About 50% of these Wnt1-Cre inactivated *Npr2* mutants developed normally as demonstrated by a nasal to anal body length and a body weight that were indistinguishable from wild type or the homozygous floxed strain (Figures 1E–J). Furthermore, grip strength in these mutant mice appeared normal (Figure 1N). However, about 50% of *Npr2*^*fl*/*fl*^*;Wnt1*^*Cre*^ mice were retarded in their development which revealed also a reduced survival rate at post-weaning stages (Figure 1F). Premature death of these mutants was accompanied by a severe malocclusion of the upper and lower incisors and intestinal distension which both affected normal nutrition (Figure S1). These mice were excluded from further testing. A malocclusion in conditional mutants of the Npr2 ligand CNP (Nakao et al., 2015) and milk retention in the stomach and intestinal distension have also been observed in the *Npr2* mutant *slw/slw* (Sogawa et al., 2010).

Analysis of sensory axon bifurcation by DiI tracing at embryonic day 12.5 or by the reporter line *Thy1-YFP-H* at postnatal day P15 indicated a complete absence of axon bifurcation at the dorsal root entry zone of the spinal cord in the conditional mutant but not in the homozygous floxed allele (Figures 1K–M). DRG axons turn either in a rostral or caudal direction at the lateral margin of the cord. In contrast, the formation of collaterals from stem sensory axons in the dorsal funiculus is not impaired in the absence of Npr2 (data not shown) indicating that this type of branch formation is not dependent on Npr2 signaling as described previously (Schmidt et al., 2007). In contrast to conditional inactivation of *Npr2* by Wnt1-Cre the use of other Cre-lines such as *trkA-Cre* or *trkC-Cre* did not induce bifurcation errors most likely due to later initiation of Cre expression with respect to Npr2 in embryonic DRG neurons (Figure S2). Overall, our data indicate that the *Npr2*^*fl*/*fl*^*;Wnt1*^*Cre*^ mutant is a suitable model to study the consequences of impaired axon bifurcation in adult mice.

### Receptive fields in the spinal cord are altered in the absence of sensory axon bifurcation

**Figure 2:**
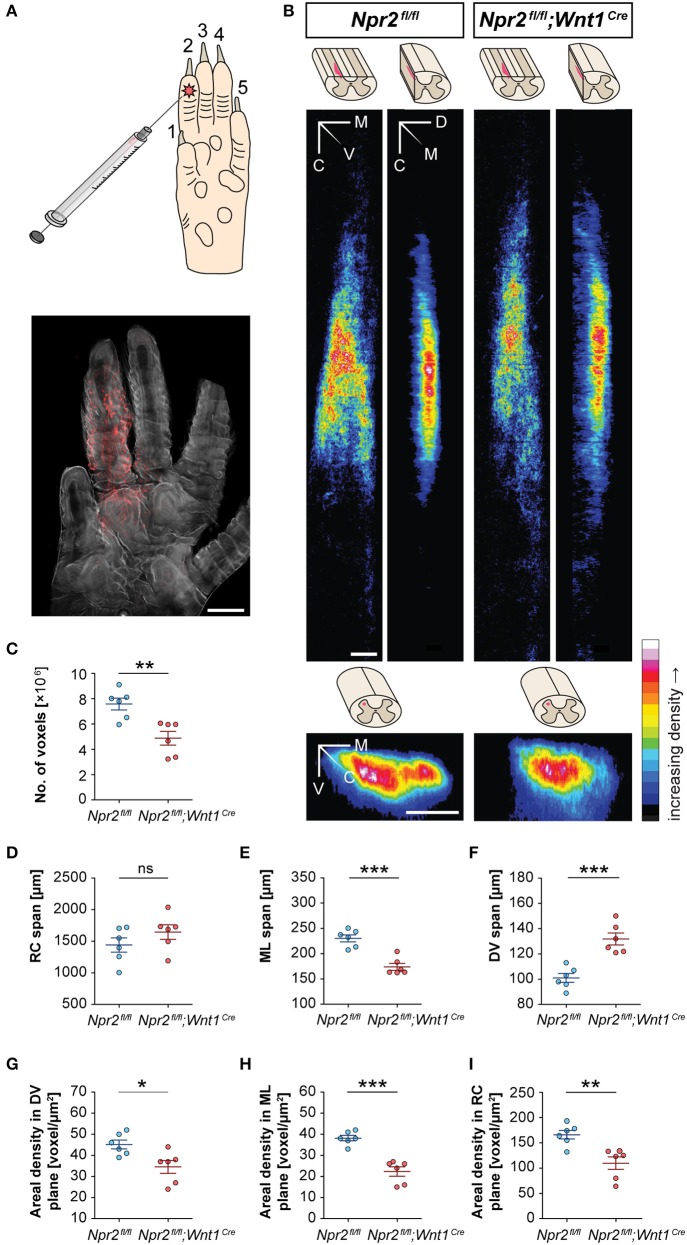
Receptive fields of spinal terminations of tactile afferents from the hindpaw in wild type and Npr2-deficient conditional mice. **(A)** Subcutaneous injection of 0.3 μl of CTB conjugated to AL594 into the second digit of the left hind paw in order to label the terminal fields of cutaneous myelinated afferents in the spinal cord dorsal horn. A fluorescent micrograph of the left hind paw is shown. Scale bar, 1 mm. **(B)** Heat maps of averaged summed projections of spinal terminal fields in *Npr2*^*fl*/*fl*^*;Wnt1*^*Cre*^ and *Npr2*^*fl*/*fl*^ control mice. Terminal fields of fibers innervating the second digit of the left hind paw appear different in *Npr2*^*fl*/*fl*^*;Wnt1*^*Cre*^ mice (*n* = 6) than in *Npr2*^*fl*/*fl*^ mice (*n* = 6) in dorsoventral, mediolateral, and rostrocaudal projections. The ImageJ color lookup table “16 Colors” was applied (see color map). The color code in each pixel denotes the number of voxels found in corresponding positions along different axes averaged across mice. Scale bars, 100 μm. **(C)** In *Npr2*^*fl*/*fl*^*;Wnt1*^*Cre*^ mice, the number of voxels representing CTB-labeled fibers is significantly lower compared to those in *Npr2*^*fl*/*fl*^ mice. **(D–F)** Morphometrics of spinal terminal fields in *Npr2*^*fl*/*fl*^*;Wnt1*^*Cre*^ and *Npr2*^*fl*/*fl*^ control mice. Spinal terminal fields of fibers innervating the second digit of the left hind paw span similarly far in the **(D)** RC dimension but in a significantly different range in the **(E)** ML and further in the **(F)** DV dimension in *Npr2*^*fl*/*fl*^*;Wnt1*^*Cre*^ (red, *n* = 6) as compared to *Npr2*^*fl*/*fl*^ (blue, *n* = 6) mice. **(G–I)** Areal densities of spinal terminal fields in *Npr2*^*fl*/*fl*^ and *Npr2*^*fl*/*fl*^*;Wnt1*^*Cre*^ mice. The areal densities of terminal fields innervating the second digit of the left hind paw digit were significantly lower in **(G)** the dorsoventral projection, **(H)** the mediolateral projection, and **(I)** the rostrocaudal projection in *Npr2*^*fl*/*fl*^*;Wnt1*^*Cre*^ (red, *n* = 6) as compared to *Npr2*^*fl*/*fl*^ (blue, *n* = 6) mice. Individual data points and mean values ± SEM are shown. Each data set was compared using a two-tailed unpaired *t*-test (^*^p < 0.05; ^**^p < 0.01; ^***^p < 0.001). CTB, cholera toxin subunit B; WT, wild type; M, medial; V, ventral; C, caudal; D, dorsal; RC, rostrocaudal; ML, mediolateral; DV, dorsoventral.

Sensory information from a large number of afferent axons converges within the spinal cord in nociceptive, mechanoreceptive or proprioceptive fields by the generation of collaterals that terminate in specific layers. Bifurcation of sensory axons at the DREZ contributes to this overall representational organization (Brown, 1981). The normal body length of the conditional *Npr2* mutant allowed us to clarify whether the absence of bifurcation affects the size or shape of termination fields of afferents from the skin. A lack of bifurcation at the DREZ predicts altered receptive fields and less overlap between different sensory modalities. Therefore, we mapped the spinal terminations of cutaneous myelinated afferents from the hind paw in wild type and *Npr2*^*fl*/*fl*^*;Wnt1*^*Cre*^ mice. The topography of primary afferent termination was visualized by transganglionic transport of fluorescently labeled cholera toxin B (CTB) subcutaneously injected into the second digit of the left hind paw of 5-week old mice (Figure 2A). CTB binds to the ganglioside GM1 which is highly expressed on axons (Kobbert et al., 2000). Injection in digit two resulted in a characteristic fork-like afferent fiber termination field in control *Npr2*^*fl*/*fl*^ spinal cords (Figure 2B). Total number of positive voxels representing CTB-labeled fibers as well as spans and area densities in dorso-ventral, medio-lateral, and rostro-caudal planes were calculated (Figures 2C–I). The total number of voxels was reduced to 64% for the 2nd digit (Figure 2C) indicating that the number of incoming sensory axons and collaterals were strongly reduced in comparison to wild type - surprisingly not to 50% as one might have been expected from the total lack of bifurcation at the DREZ. This observation might be explained by an increase in the density of collaterals protruding from the stem axons or by an increase in the terminal branching of collaterals in the mutant. Interestingly, the dorsoventral span of fluorescence intensity was increased to about 131% of control for the 2nd digit in the mutant whereas the medial-to-lateral distribution of terminal field was decreased by 25%. No difference was observed in the rostral-to-caudal span of the projection (Figures 2D–F). The density, the voxels per area, decreased to 66 and 58% in the rostrocaudal and in the mediolateral direction, respectively, whereas the dorsoventral direction decreased only to 76% (Figures 2G–I) for digit two. In summary, the absence of bifurcation caused decreased projection intensities together with a reduction in the mediolateral span as well as an increase in the dorsoventral extension of sensory afferent termination fields in the spinal cord.

### Balance and motor coordination is not impaired in the absence of sensory axon bifurcation

**Figure 3:**
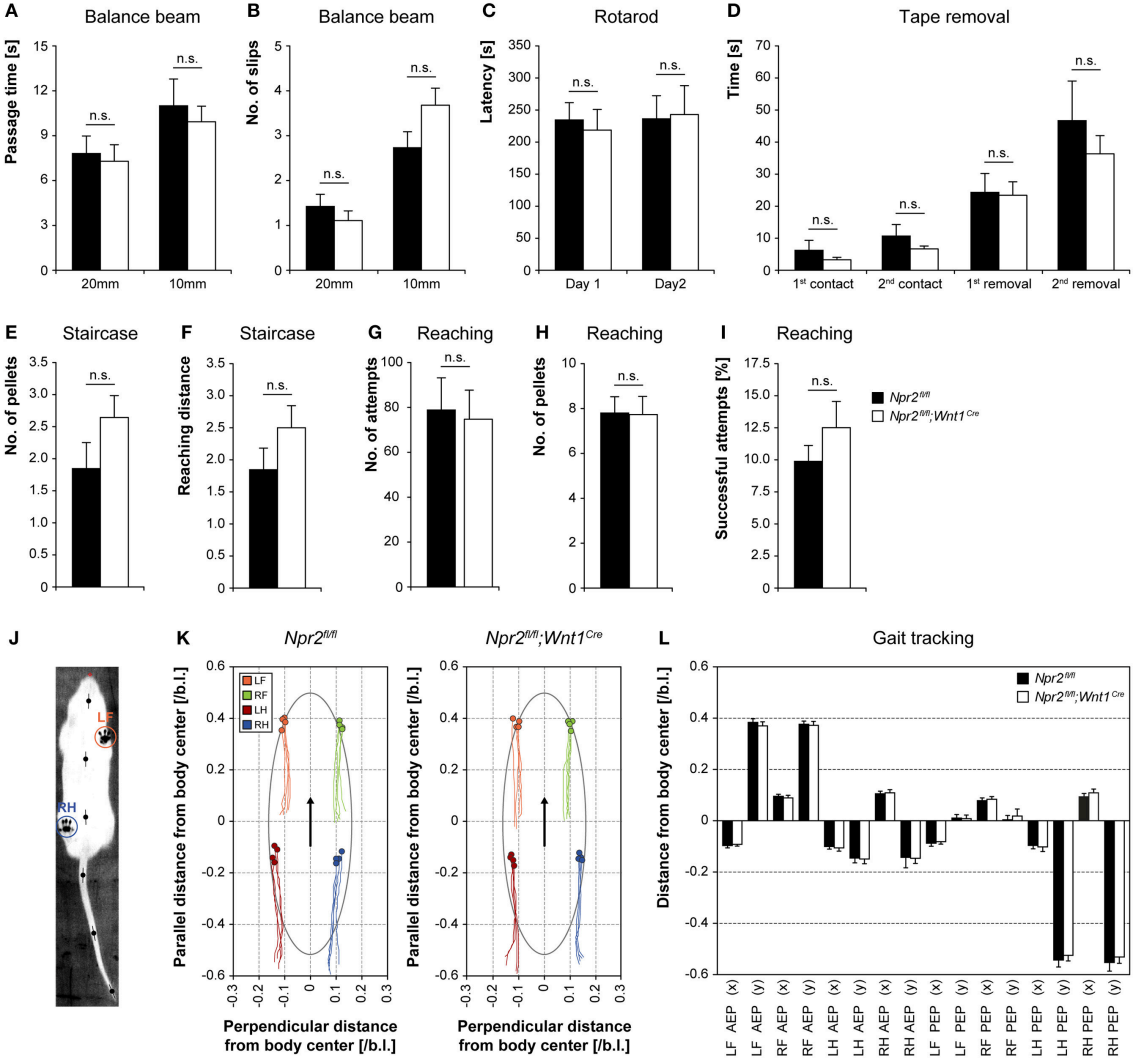
Analysis of balance and motor coordination in the absence of *Npr2*^*fl*/*fl*^*;Wnt1*^*Cre*.^ Balance and motor coordination in Npr2-deficient mice was not altered in the absence of DRG axon bifurcation using the **(A)** beam walking test (*n* = 13 *Npr2*^*fl*/*fl*^ and 14 *Npr2*^*fl*/*fl*^*;Wnt1*^*Cre*^), passage time and **(B)** number of slips, **(C)** rotarod performance test (*n* = 5), **(D)** tape removal test (*n* = 13 *Npr2*^*fl*/*fl*^ and 14 *Npr2*^*fl*/*fl*^*;Wnt1*^*Cre*^), **(E)** staircase test, no. of pellets and **(F)** reaching distance (*n* = 13 *Npr2*^*fl*/*fl*^ and 14 *Npr2*^*fl*/*fl*^*;Wnt1*^*Cre*^), **(G)** food grasping and reaching, number of attempts, **(H)** number of pellets and **(I)** successful attempts (*n* = 10 *Npr2*^*fl*/*fl*^ and 15 *Npr2*^*fl*/*fl*^*;Wnt1*^*Cre*^), **(J)** walking track analysis using the MouseWalker, **(K)** Plots of stand traces of free walking animals and **(L)** summary of paw positioning in *Npr2*^*fl*/*fl*^ (*n* = 4) and *Npr2*^*fl*/*fl*^*;Wnt1*^*Cre*^ (*n* = 4) are shown. AEP, anterior extreme position; PEP, posterior extreme postion; L, left; R, right; F, forepaw; H, hind paw; error bar: SEM. No significant differences were observed between *Npr2*^*fl*/*fl*^ and *Npr2*^*fl*/*fl*^*;Wnt1*^*Cre*^ mice in any of these tests.

Since axon bifurcation is compromised in all DRG neurons of Npr2-deficient mice we asked whether proprioception might be impaired. Therefore, balance and motor coordination in *Npr2*^*fl*/*fl*^*;Wnt1*^*Cre*^ mice was examined using the balance beam test, rotarod, staircase assay, food grasping and reaching assay, and walking track analysis (MouseWalker). Balance beam and rotarod measure the ability to perform complex and coordinated movements. Npr2-deficient mice did not show a reduced latency to fall off or to slip off the beam (Figures 3A,B) or to fall off the rotating rod (Figure 3C). Similarly, no deficits were observed in the staircase and reaching and grasping test (Figures 3E–I), indicating that motor coordination was not altered in the absence of sensory axon bifurcation. To assess sensory and motor abilities related to the forepaw and mouth coordination the adhesion removal test was performed. Adhesive tape was fixed gently to the hairless parts of the forepaws and the time to first contact and the removal of both adhesive strips was recorded. Control mice and Npr2 mutants made regular attempts to remove the tape (Figure 3D). To ask whether the absence of sensory axon bifurcation interferes with the control of locomotion we applied the MouseWalker system which allows a comprehensive and quantitative description of parameters of freely walking Npr2-deficient mice (Figures 3J–L).

In summary, no deficits were observed in these behavioral assays in conditional *Npr2* mutants (Figure 3). Npr2-deficient mutants possess considerable coordination capabilities.

### Heat sensation is impaired in the absence of sensory axon bifurcation in *Npr2^*fl*/*fl*^;Wnt1^*Cre*^* mice

To assess the consequences of impaired axonal bifurcation of sensory neurons for pain processing *in vivo*, we analyzed the nociceptive behavior of *Npr2*^*fl*/*fl*^*;Wnt1*^*Cre*^ and respective control mice in various animal models of pain. We tested both male and female mice, but no significant effects of sex were detected in any assay.

**Figure 4:**
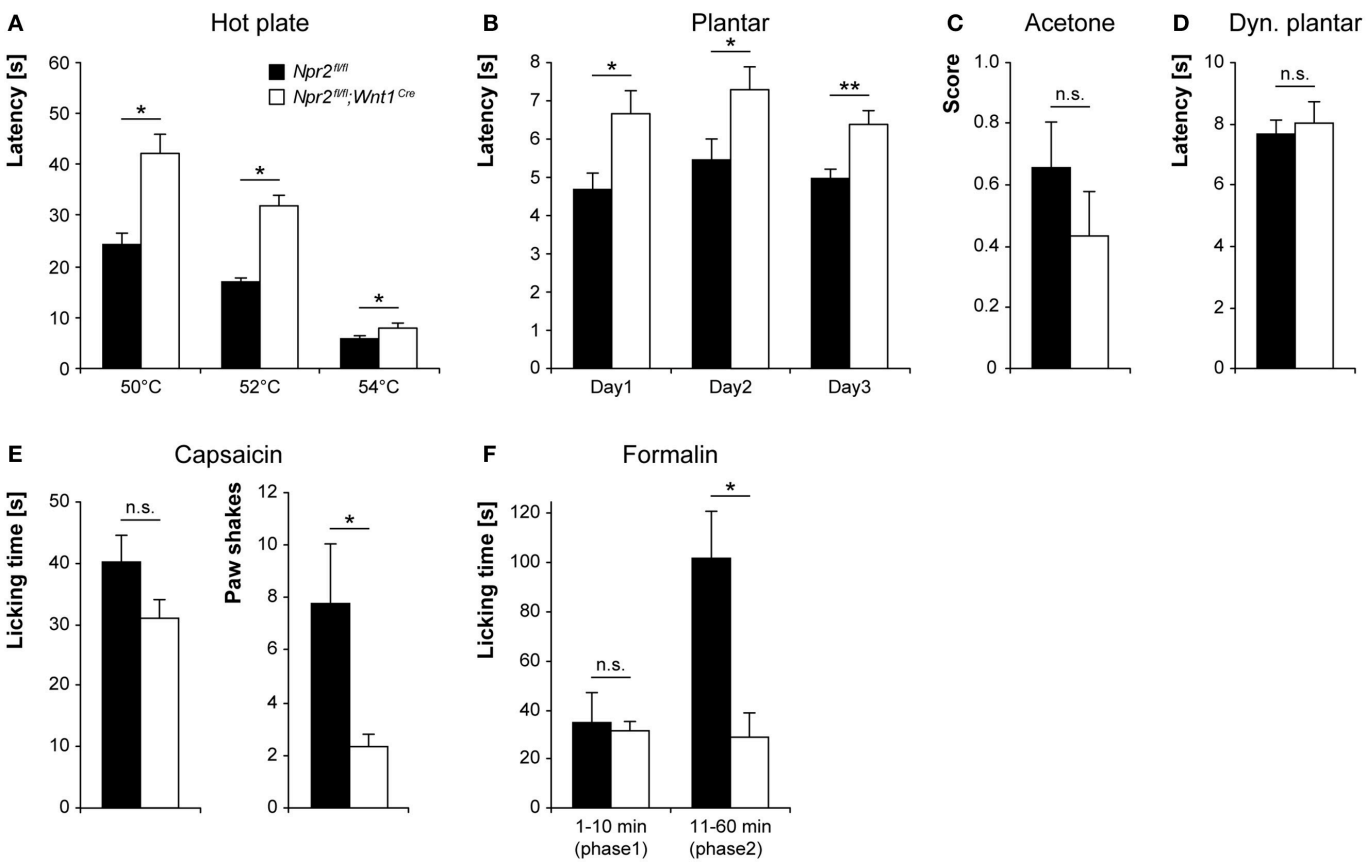
Pain behavior in *Npr2*^*fl*/*fl*^*;Wnt1*^*Cre*^ and littermate control mice. **(A)** Thermal heat pain was assessed on a 50, 52, and 54°C hot plate (*n* = 15–16; ^*^p < 0.05; power 97, 99, and 53% for 50, 52, and 54°C, respectively). **(B)** Thermosensation by the Hargreaves method (Plantar test) performed at 3 consecutive days (*n* = 8; ^*^p < 0.05 and ^**^p < 0.01; power 70, 56, and 85% for day 1, 2, and 3, respectively). **(C)** Cold pain was investigated using acetone-evoked evaporative cooling (*n* = 15–16, power 17%). **(D)** Mechanical sensitivity was measured using a dynamic plantar test (*n* = 11, power 11%). **(E,F)** The nocifensive response to chemical pain stimuli was determined by injection of 5 μg capsaicin (*n* = 9, power 36 and 55% for licking and paw shakes, respectively) or 0.5% formalin (*n* = 5, power 5 and 84% for phase 1 and 2, respectively) into a hindpaw. ^*^p < 0.05.

We first determined the acute pain thresholds for noxious thermal stimuli in *Npr2*^*fl*/*fl*^*;Wnt1*^*Cre*^ and littermate control mice using the hot plate test (50–54°C). *Npr2*^*fl*/*fl*^*;Wnt1*^*Cre*^ mice displayed typical nocifensive behaviors (shaking or licking of the hindpaws or jumping off the plate), but at all tested temperatures they did so at consistently longer latencies than control mice (Figure 4A). Paw withdrawal responses to radiant heat (Hargreaves method) were also significantly increased in *Npr2*^*fl*/*fl*^*;Wnt1*^*Cre*^ mice as compared to control mice (Figure 4B). In contrast to the impaired heat sensitivity, responses to acetone-evoked evaporative cooling were normal in *Npr2*^*fl*/*fl*^*;Wnt1*^*Cre*^ mice in the group tested (Figure 4C; *p* = 0.295). Moreover, mechanical sensitivity of the hindpaw assessed using a dynamic plantar aesthesiometer was similar in *Npr2*^*fl*/*fl*^*;Wnt1*^*Cre*^ and control mice (Figure 4D). Together, these data suggest that the loss of axonal bifurcation impairs the rapid response to avoid noxious heat, whereas behavioral thresholds and response latencies to cold or mechanical stimuli were not affected.

We next examined the tonic pain behavior in *Npr2*^*fl*/*fl*^*;Wnt1*^*Cre*^ and control mice after injection of capsaicin or formalin into a hindpaw. The capsaicin injection (5 μg) resulted in licking and shaking the injected paw in both genotypes. Whereas, the licking behavior was not statistically different between groups (*p* = 0.104), the number of paw shakes was significantly reduced in *Npr2*^*fl*/*fl*^*;Wnt1*^*Cre*^ mice compared to control mice in the test group (Figure 4E). Injection of 0.5% formalin into a hindpaw resulted in the typical biphasic paw licking. In the first phase (1–10 min), which results from acute activation of nociceptors, the licking behavior of *Npr2*^*fl*/*fl*^*;Wnt1*^*Cre*^ and control littermates was comparable (Figure 4F). However, the formalin-induced paw licking behavior was significantly reduced in *Npr2*^*fl*/*fl*^*;Wnt1*^*Cre*^ mice in the second phase (11–60 min), which involves a combination of ongoing afferent firing and central sensitization (Shields et al., 2010). These data indicate that nociception induced by the chemical irritants capsaicin or formalin are impaired by the loss of axonal bifurcation. Consistent with these results are our previously published electrophysiological recordings on global *Npr2* and *CNP* knockouts which revealed a reduced number of neurons in the dorsal spinal cord responding to capsaicin (Schmidt et al., 2007, 2009).

### Npr2 expression in DRGs declines postnatally

**Figure 5:**
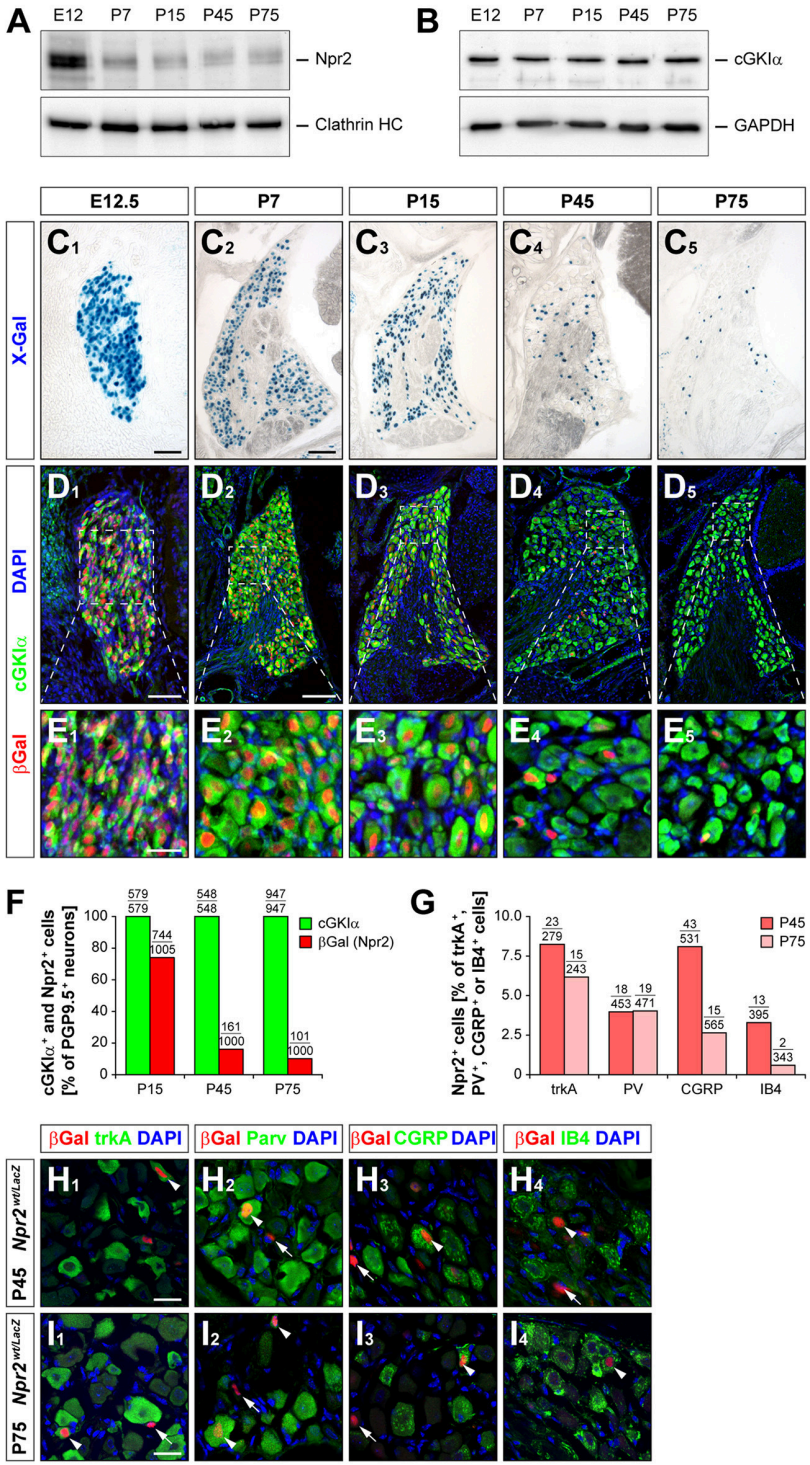
Expression of Npr2 and cGKIα in DRGs at different developmental stages. **(A,B)** Western blot of membrane or cytosolic fractions of DRGs using antibodies to Npr2 or to cGKIα, respectively. The heavy chain of clathrin or GAPDH served as loading control. **(C–E)** Sections through thoracic DRGs at different stages. Localization of anti-βGal staining indicating Npr2-expression is demonstrated in the *Npr2*^*wt*/*LacZ*^-mouse reporter and localization of cGKIα by a polyclonal antibody to mouse cGKIα. Scale bars in **(C**_1_**,D**_1_**)**, 50 μm; in **(C**_2_**-C**_5_**,D**_2_**-D**_5_**)**, 100 μm; in **(E)** 25 μm. **(F)** Quantification of Npr2-positive and cGKIα-positive neurons in DRGs counterstained with an antibody to PGP9.5 at different stages. **(G–I)** A small proportion of Npr2-positive DRG neurons at P45 and P75 express trkA, parvalbumin, CGRP or IB4. Scale bars in **(H,I)**, 20 μm.

In addition to its role in sensory axon bifurcation Npr2 could potentially have an acute function in the processing of sensory information for example in noxious heat perception or nociception induced by chemical irritants. Furthermore, the second messenger cGMP and cGKI contribute to the processing of inflammatory and neuropathic pain in the spinal cord (Tegeder et al., 2004; Schmidtko et al., 2008, 2009; Luo et al., 2012). To further explore whether the measured deficits in pain behaviors in *Npr2* mutants are caused by the absence of sensory axon bifurcation or by changes in primary acute pain perception, we studied the developmental expression profile of Npr2 in DRGs by histochemical determination of β-galactosidase activity in cryostat sections from the *Npr2*^*wt*/*lacZ*^ reporter mouse and by Western blot analysis of tissue extracts of DRGs (Ter-Avetisyan et al., 2014). Consistent with our previous *in situ* hybridization data we observed that all DRG neurons strongly express Npr2 at early embryonic stages when sensory axons enter the spinal cord (Figures 5A, C–E). At postnatal stages, however, the level and the number of Npr2 expressing cells gradually decreases in DRGs and at post-natal day 75 only 10% were positive for Npr2. Similar results were obtained by analyzing red fluorescent protein expression in DRGs at P75 which was induced by crossing the *Rosa26-tdTomato* reporter mouse line with *Npr2-CreERT2* and tamoxifen application at 3 consecutive days (P71, P72, and P73) (data not shown). Further analysis revealed that Npr2 is not confined to a specific subset of DRG neurons. It is expressed in a small group of trkA-, parvalbumin-, CGRP-, or IB4-expressing DRG neurons. The percentage of Npr2-positive cells in these subpopulations - except for parvalbumin - decreased during maturation (Figures 5F–I).

### Cutaneous C-fiber thermal and mechanical sensitivity is normal in Npr2 mutants

**Figure 6:**
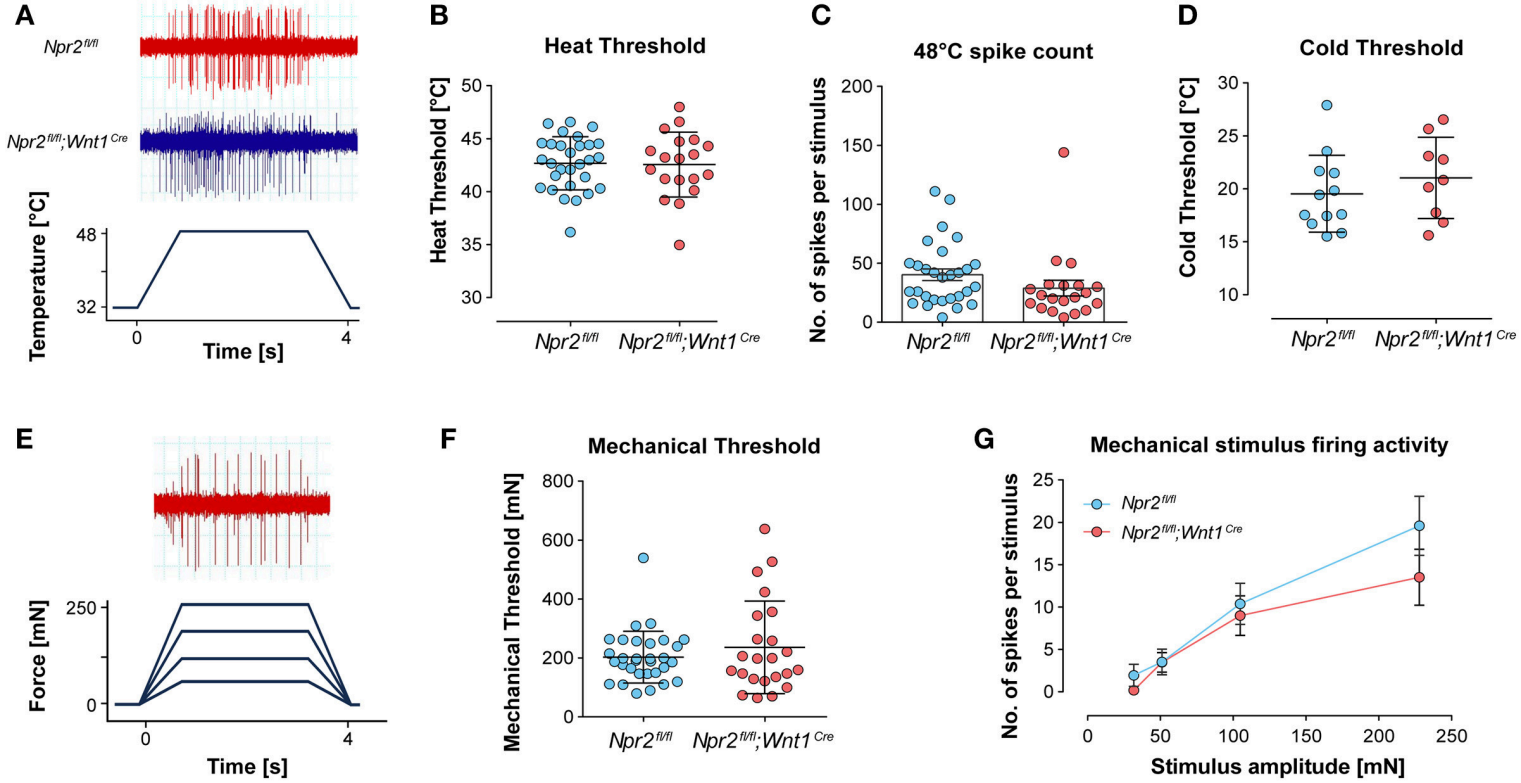
Responses of cutaneous hindpaw C-fibers to mechanical and thermal stimuli. Hindpaw cutaneous C-fibers with receptive fields on hairy foot skin were recorded from *Npr2*^*fl*/*fl*^*;Wnt1*^*Cre*^ mice and littermate controls using the *ex vivo* skin nerve preparation. **(A)** Example traces of C-fiber firing activity recorded from *Npr2*^*fl*/*fl*^*;Wnt1*^*Cre*^ mice and littermate controls in response to heat stimulation. **(B)** Heat thresholds of C-fibers were investigated using a 32–48°C 15 s heat ramp protocol. **(C)** The total number of spikes were quantified over the 4 s heat stimulus shown in **(A)**. **(D)** Cold thresholds were investigated using a 32–12°C 15 s heat ramp protocol. **(E)** The total numbers of spikes were quantified over the 4 s heat stimulus shown in **(A)**. **(F)** Mechanical thresholds of C-fibers. **(G)** Mechanical stimulus-evoked firing activity of C-fibers was quantified in response to four ramp and hold stimuli and compared between groups (see also Table S1).

Co-stainings indicated that a small percentage of Npr2-positive DRG neurons at P75 express parvalbumin or trkA; therefore, it is possible that changes in peripheral nociceptor function may underlie altered heat sensitivity in *Npr2*^*fl*/*fl*^*;Wnt1*^*Cre*^ mice. To test this, extracellular recordings from single C-fiber units in the hindpaw skin were performed using the *ex vivo* skin nerve preparation. C-fiber responses to heat, cold and mechanical stimulation of receptive fields were characterized in a total of 24 thermal-responsive C-fibers in *Npr2*^*fl*/*fl*^*;Wnt1*^*Cre*^ mice, and 33 units in littermate control mice (Table S1). Example heat-evoked firing activity of single C-fibers in *Npr2*^*fl*/*fl*^*;Wnt1*^*Cre*^ mice and littermate controls are shown in Figure 6A.

C-fiber heat thresholds, defined as the temperature required to evoke the first action potential, did not differ between *Npr2*^*fl*/*fl*^*;Wnt1*^*Cre*^ mice and littermate controls (Figure 6B). Similarly, there was no significant difference in the number of action potentials in C-fibers during heat stimulation between *Npr2*^*fl*/*fl*^*;Wnt1*^*Cre*^ mice and littermate controls in response to suprathreshold heat stimulation (Figure 6C, exemplified in Figure 6A). Cold thresholds of C-fibers were also not different between *Npr2*^*fl*/*fl*^*;Wnt1*^*Cre*^ mice and littermate controls (Figure 6D). Mechanical thresholds and suprathreshold mechanical stimulus-evoked firing activity of C-fibers also did not differ between *Npr2*^*fl*/*fl*^*;Wnt1*^*Cre*^ mice and littermate controls (Figures 6E,F). Example mechanical-evoked firing activity of a C-fibers recorded from a *Npr2*^*fl*/*fl*^*;Wnt1*^*Cre*^ mouse is shown in Figure 6G. These data indicate that thermal and mechanical coding properties of cutaneous C-fibers are not functionally impaired in *Npr2* mutant mice.

### Conditional inactivation of cGKI abolishes sensory axon bifurcation and partially mimics the phenotype of *Npr2* mutants in pain-related behavior

cGKI is an essential signaling component downstream of Npr2 to induce sensory axon bifurcation as shown previously (Schmidt et al., 2002, 2007; Zhao et al., 2009). However, the developmental expression profile of cGKI is distinct from Npr2. At early embryonic stages when sensory axons are bifurcating in the spinal cord a complete overlap between Npr2 and cGKIα in all sensory neurons was observed (Figures 5B,D,E). In contrast to Npr2, cGKIα remains expressed in all sensory neurons also at mature stages suggesting that its function might be regulated by other cGMP synthesizing enzymes than by Npr2 in adult mice.

**Figure 7:**
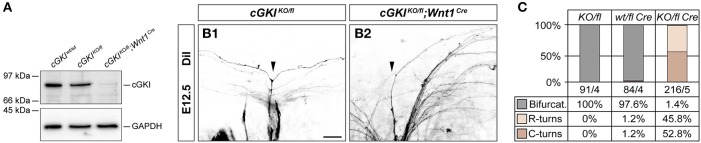
Conditional inactivation of *cGKI*. **(A)** Conditional inactivation by Wnt1-Cre results in the complete absence of cGKI protein in embryonic DRGs as revealed by Western blot detection of cGKI in cytosolic fractions of E13.5 DRG from *cGKI*^wt/wt^, *cGKI*^*KO*/*fl*^, and *cGKI*^*KO*/*fl*^*;Wnt1*^*Cre*^ mice. Equal loading was demonstrated by reprobing of the blot with an antibody to GAPDH. **(B,C)** Complete absence of sensory axon bifurcation in *cGKI*^*KO*/*fl*^*;Wnt1*^*Cre*^ and quantification. The numbers of counted axons and embryos are indicated below the columns. Scale bar, 25 μm.

The absence of cGKI in a constitutive knockout does not interfere with bone growth but shows deficits in the regulation of smooth muscle contraction with vascular and intestinal dysfunctions which cause pre-mature death (Hofmann et al., 2006). Behavioral testing therefore also required conditional inactivation of cGKI in DRG neurons, which was obtained by a floxed allele of the *cGKI* gene (Wegener et al., 2002) and using *Wnt1-Cre* as described for *Npr2*. This resulted in a complete loss of cGKI protein in DRGs as indicated by Western blotting using antibodies to cGKI (Figure 7A). Consequently, sensory axons failed to form bifurcational branches at the DREZ (Figures 7B,C). In contrast to conditional Npr2 mutants, no decrease in the survival rate or any problems on the health status of 23 inspected *cGKI*^*KO*/*fl*^*;Wnt1*^*Cre*^ animals in which cGKI was conditionally inactivated was observed. In some tissues Npr2-induced cGMP signals are mediated by the cGKII instead of cGKI which might explain these phenotypic differences on the survival rate between cGKI and Npr2.

**Figure 8:**
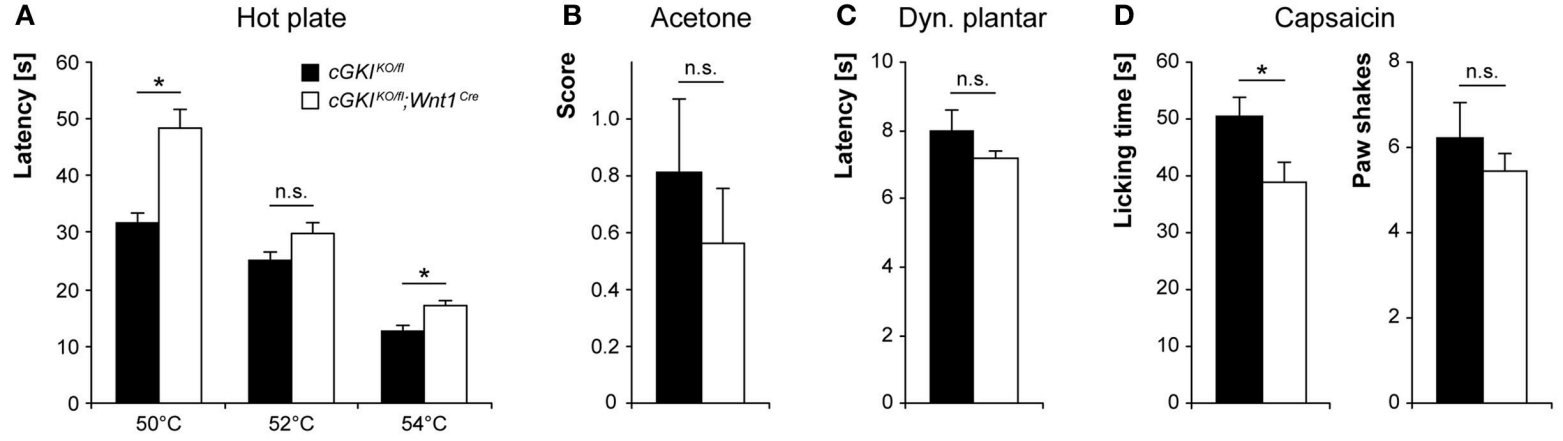
Pain behavior in *cGKI*^*KO*/*fl*^*;Wnt1*^*Cre*^ and littermate control mice. **(A)** Thermal heat pain was assessed on a 50, 52, and 54°C hot plate (*n* = 16; ^*^p < 0.05; power 99, 47, and 90% for 50, 52, and 54°C, respectively). **(B)** Cold pain was investigated using acetone-evoked evaporative cooling (*n* = 16, power 11%). **(C)** Mechanical sensitivity was measured using a dynamic plantar test (*n* = 12, power 20%). **(D)** The nocifensive response to a chemical pain stimulus was determined by injection of 5 μg capsaicin (*n* = 9, power 61% for licking and 12% for paw shakes) into a hindpaw. ^*^p < 0.05.

To estimate whether the observed Npr2-dependent changes in pain processing are mediated by a Npr2/cGMP/cGKI signaling pathway, we analyzed the nociceptive behavior of *cGKI*^*KO*/*fl*^*;Wnt1*^*Cre*^ and littermate control mice (*cGKI*^*KO*/*fl*^). In the hot plate test, *cGKI*^*KO*/*fl*^*;Wnt1*^*Cre*^ mice demonstrated reduced noxious heat sensation (Figure 8A), albeit with significant differences compared to control mice only at 50 and 54°C. In the acetone test (Figure 8B) and dynamic plantar test (Figure 8C), the responses to cold and mechanical stimuli were normal in *cGKI*^*KO*/*fl*^*;Wnt1*^*Cre*^ mice. After capsaicin injection into a hindpaw, the paw licking behavior of *cGKI*^*KO*/*fl*^*;Wnt1*^*Cre*^ mice was reduced as compared to *cGKI*^*KO*/*fl*^ control mice (Figure 8D). Altogether, both *Npr2*^*fl*/*fl*^*;Wnt1*^*Cre*^ and *cGKI*^*KO*/*fl*^*;Wnt1*^*Cre*^ mice demonstrated impaired responses to noxious heat and chemical stimuli but normal responses to cold and mechanical stimuli, suggesting that the loss of bifurcation causes specific deficits in nociceptive processing.

## Discussion

Previous studies using knockout mice and axon tracing methods have implicated a cGMP signaling cascade composed of the ligand CNP, the receptor guanylyl cyclase Npr2 and the cGMP-dependent kinase I in bifurcation of DRG and cranial sensory ganglia (Schmidt and Rathjen, 2010; Gibson and Ma, 2011; Ter-Avetisyan et al., 2014). Since constitutive knockouts of *Npr2* and *cGKI* are not suitable for further anatomical and behavioral studies on the consequences of the primary defect, the branching error at the DREZ, we generated mouse models that allowed an inactivation of Npr2 or cGKI specifically in embryonic DRGs at very early developmental stages before sensory axons enter the spinal cord. The *Wnt1-Cre* line was suitable to completely inactivate Npr2 or cGKI resulting in a complete lack of bifurcation for all DRG axons as previously described for the constitutive knockouts. Other Cre lines such as *trkA-Cre* or *trkC-Cre* did not induce bifurcation errors most likely by mediating recombination at relatively late stages of development and therefore cannot be used to assess a role of axonal bifurcation. The conditional inactivation of Npr2 by *Wnt1-Cre* allowed us to study the behavioral consequences and additional anatomical changes in the termination fields in the spinal cord caused by the disturbed axon bifurcation at the DREZ.

Due to the lack of axon bifurcation a reduction in the number of collaterals originating from primary afferents of DRG neurons within the spinal cord is not unexpected. However, the observed reduction to 64% of control values and not to 50% when total voxels numbers were summed up points to compensatory mechanisms such as an increase in the density of collaterals on stem axons, increased terminal branching of collaterals in spinal target areas or increased cell number in DRGs. The latter can be excluded since numbers were unchanged in the absence of cGMP signaling (Schmidt et al., 2002). Topographic representation of the sensory periphery within the spinal cord is of fundamental importance for information processing (Brown et al., 1991; Florence et al., 1991; Levinsson et al., 2002; Schouenborg, 2004; Granmo et al., 2008). Our work reported here showed that not only a quantitative reduction of incoming fibers but also a change in the pattern in the termination fields was detected in the absence of bifurcation. Npr2-deficiency caused an increase of the dorsoventral span to 131% of the termination field of digit two of the hindpaw whereas the mediolateral extension was narrowed by 25%. In principle pre- and postsynaptic mechanisms contribute to the formation of terminal fields. In the *Npr2* mutants the balance between these interacting structures is disordered in the spinal cord. Our observations suggest that the incoming sensory axons and most likely less the postsynaptic neurons within the spinal cord might be the causative determinants on the development of these modified fields. Less sensory collaterals compete for postsynaptic neurons which might result in an increase of terminal branching in dorsoventral direction. In other words, in *Npr2* mutants collaterals project not just to the normal zone of responding postsynaptic cells. The opposite is found for the mediolateral extension and no change was observed for the rostrocaudal span. The latter was expected since the longitudinal growth of the stem axons was not diminished in the absence of cGMP signaling (Schmidt et al., 2002).

In behavioral assays responses to noxious heat and chemical stimuli were impaired by the loss of axonal bifurcation either by the absence of Npr2 or cGKI which is in line with our previously published patch clamp recordings in Npr2- or CNP-deficient slices of the dorsal horn of the spinal cord. A reduced number of responding cells upon capsaicin treatment was measured in both mutants in comparison to wild type tissue, whereas synaptic transmission *per se* was not affected (Schmidt et al., 2007, 2009). In addition, in a recent study using global *Npr2* mutants - in which, however, bone growth is disturbed - deficits in the auditory system have been described (Lu et al., 2014). Although the downstream effector cGKI shows a widespread pattern of localization in DRGs that is distinct from the more restricted expression of Npr2 in mature stages, similar - however not fully identical - deficits in nociception were observed in the behavioral tests upon the conditional ablation of cGKI. Its widespread localization in mature DRG neurons, its multiple phosphorylation targets (Hofmann and Wegener, 2013) and its involvement in synaptic potentiation in spinal neurons (Luo et al., 2012) might contribute to the differences in the licking time vs. paw shakes that were observed upon capsaicin treatment in *Npr2* and *cGKI* conditional mutants. Our interpretation that the deficits in noxious heat perception in *Npr2* and *cGKI* mutated mice were caused by the absence of axon bifurcation are in line with studies on a mouse mutant in which cGKI was inactivated by a Cre recombinase under control of the Na_v_1.8 promoter (*SNS-Cre*). These mice do not show axon bifurcation defects at the DREZ due to the expression of the Cre recombinase after the ingrowth of sensory axons. They responded normally to noxious heat as measured by paw withdrawal to radiant heat (Hargreaves method) (Luo et al., 2012). In contrast to nociception, balance and motor performance appear normal in the conditional absence of Npr2-mediated bifurcation suggesting that pain processing might be more sensitive to a lack of bifurcation than proprioception. Moreover, nociceptors and other chemoreceptors have small-diameter axons which are either unmyelinated or thinly myelinated and therefore conduct action potentials more slowly. In contrast, proprioception, which is mediated by myelinated axons with a large diameter, might be more flexible due to fast conductance of action potentials and might therefore adjust more rapidly. It is also conceivable that elaborate compensatory mechanisms are implemented in the proprioceptive system to reorganize neuronal circuits in the absence of bifurcation. These compensational mechanisms might be less active in the nociceptive system.

It also cannot be excluded that the significant differences observed between nociception and proprioception in the absence of bifurcation might be related to the experimental systems that were applied. Pain was always analyzed by paw withdrawal reactions and a time delay in responses was measured (Weng and Schouenborg, 1998). In such a system pain perception needs to be transferred to muscle activity and this might require axon bifurcation at the DREZ to be well-coordinated and effectively executed. Further tactile feedback might be important for the nociceptive withdrawal reflex system. In addition accurate pain signaling might involve the integration of sensory information from several afferent subtypes and might require a complex crosstalk between spinal interneurons which might not be fully developed in the absence of axon bifurcation.

An acute or a morphological role of Npr2 on interneurons in the spinal cord or in the brain which might contribute to the measured behavioral deficits cannot currently be fully excluded. However, we never observed any changes in the arrangement of specific cell layers in the spinal cord, in the overall growth of proprioceptive collaterals or any synaptic deficits in the absence of Npr2 or CNP (Schmidt et al., 2007, 2009). Bifurcation errors of sensory axons were the only changes in the absence of the CNP/Npr2/cGKI signaling cascade we observed in the nervous system so far. Our electrophysiological recordings on the C-fibers in the nerve-skin preparation make it unlikely that Npr2 has an acute function in the conduction of sensory information. Nevertheless, a detailed pattern of expression of Npr2 in the developing and mature brain and spinal cord remains to be established including investigations on a co-localization with cGKI and Wnt1-Cre recombinase. In the future selective inactivation of Npr2 in spinal interneurons but not in DRG neurons might further substantiate or relativize our interpretations on the deficits caused by axonal bifurcation.

In humans, loss-of-function mutations in the *NPR2* gene cause a skeletal dysplasia, termed acromesomelic dysplasia type Maroteaux (AMDM) with an extremely short and disproportionate stature (Bartels et al., 2004). AMDM is a rare autosomal-recessive genetic disorder with a prevalence of one out of 1,000,000. Point mutations leading to single amino acid exchanges were found throughout the NPR2 protein including the ligand binding, kinase homology or guanylyl cyclase domain (for a synopsis of human *NPR2* mutations see Vasques et al., 2014). A number of these *NPR2*-missense mutations resulted in retention of the protein in the endoplasmic reticulum and poor targeting of the protein to the plasma membrane (Hume et al., 2009; Vasques et al., 2013; Amano et al., 2014; Wang et al., 2016) while others reached the cell surface (Dickey et al., 2016). Radiographic images demonstrated abnormal growth plates and short bones in the limbs detectable by two years of age. Carrier parents of individuals with AMDM and heterozygous mutations in *NPR2* are associated with a slight reduction in height in comparison to the population average (Olney et al., 2006; Vasques et al., 2013; Hisado-Oliva et al., 2015) which was also found for mice heterozygous for *Npr2* (Tamura et al., 2004). Heterozygosity in *Npr2* or *cGKI* does not affect axonal bifurcation in mice (Schmidt et al., 2007). In contrast, *NPR2* gain-of-function mutations result in an overgrowth syndrome (Miura et al., 2014). Currently, it can only be hypothesized whether the absence of the Npr2-mediated cGMP signaling axis in DRG neurons causes branching errors of sensory axons within the spinal cord in these patients. In addition, detailed neurological tests which might reveal neurological deficits have not been performed in AMDM patients so far. However, studies on mutations in rodents on axonal guidance factors have established a good correlation between animal models and human diseases (Jen et al., 2004; Engle, 2010; Srour et al., 2010; Depienne et al., 2011; Nugent et al., 2012; Chedotal, 2014). Our data using mouse genetics indicated deficits in noxious heat perception and nociception induced by the chemical irritants capsaicin or formalin. These observations might provide a framework for future studies to characterize neurological qualities of human patients with mutations in the *NPR2* gene.

## Author contributions

PT, JH, JP, OD, FS, GT-A, SP, SF, FR, and HS performed experiments. Conception, drafting, approval, and agreement to be accountable for all aspects of the work in ensuring that questions related to the accuracy or integrity of any part of the work are appropriately investigated and resolved: PT, JH, JP, OD, AS, FS, GL, GT-A, YW, SP, SF, RF, FR, and HS.

### Conflict of interest statement

The authors declare that the research was conducted in the absence of any commercial or financial relationships that could be construed as a potential conflict of interest.

## Supplementary material

Figure S1 Severe malocclusions of P22 *Npr2*^*fl*/*fl*^*;Wnt1*^*Cre*^ mice which prevents normal nutrition and correlated with premature death. Such animals have been excluded from behavioral testing.

Figure S2 No bifurcation errors of DRG axons were observed when the *Npr2-flox* line was crossbred with *trkA-Cr*e or *trkC-Cre*. **(A)** Example of a DiI-labeled axon from E12.5 *Npr2*^*fl*/*fl*^*;trkA*^*Cre*^. Scale bar, 50 μm. **(B)** Quantification of bifurcation behavior of DRG axons in *Npr2*^*fl*/*fl*^ and *Npr2*^*fl*/*fl*^*;trkA*^*Cre*^ embryos at E12.5. Numbers below the columns indicate counted axons and embryos. **(C)** Dorsolateral view of DRG axons at the dorsal root entry zone of a *Npr2*^*fl*/*fl*^*;trkC*^*Cre*^*;Thy1*^*YFP*−*H*^ mouse at P21. Bar, 100 μm.

Table S1 C-fiber *ex vivo* skin nerve preparation recorded from the hindpaw hairy skin. C-mechanoheat (CMH), C-mechanoheatcold (CMHC), and C-mechanocold (CMC) fibers were recorded from *Npr2*^*fl*/*fl*^*;Wnt1*^*Cre*^ mice and littermate controls. Mean CVs are shown ± SEM.
