# Supplementary figures and images for: The Absence of Sensory Axon Bifurcation Affects Nociception and Termination Fields of Afferents in the Spinal Cord
Source: Front Mol Neurosci. 2018 Feb 8;11:19. doi: 10.3389/fnmol.2018.00019 (PMC5809486; doi:10.3389/fnmol.2018.00019)

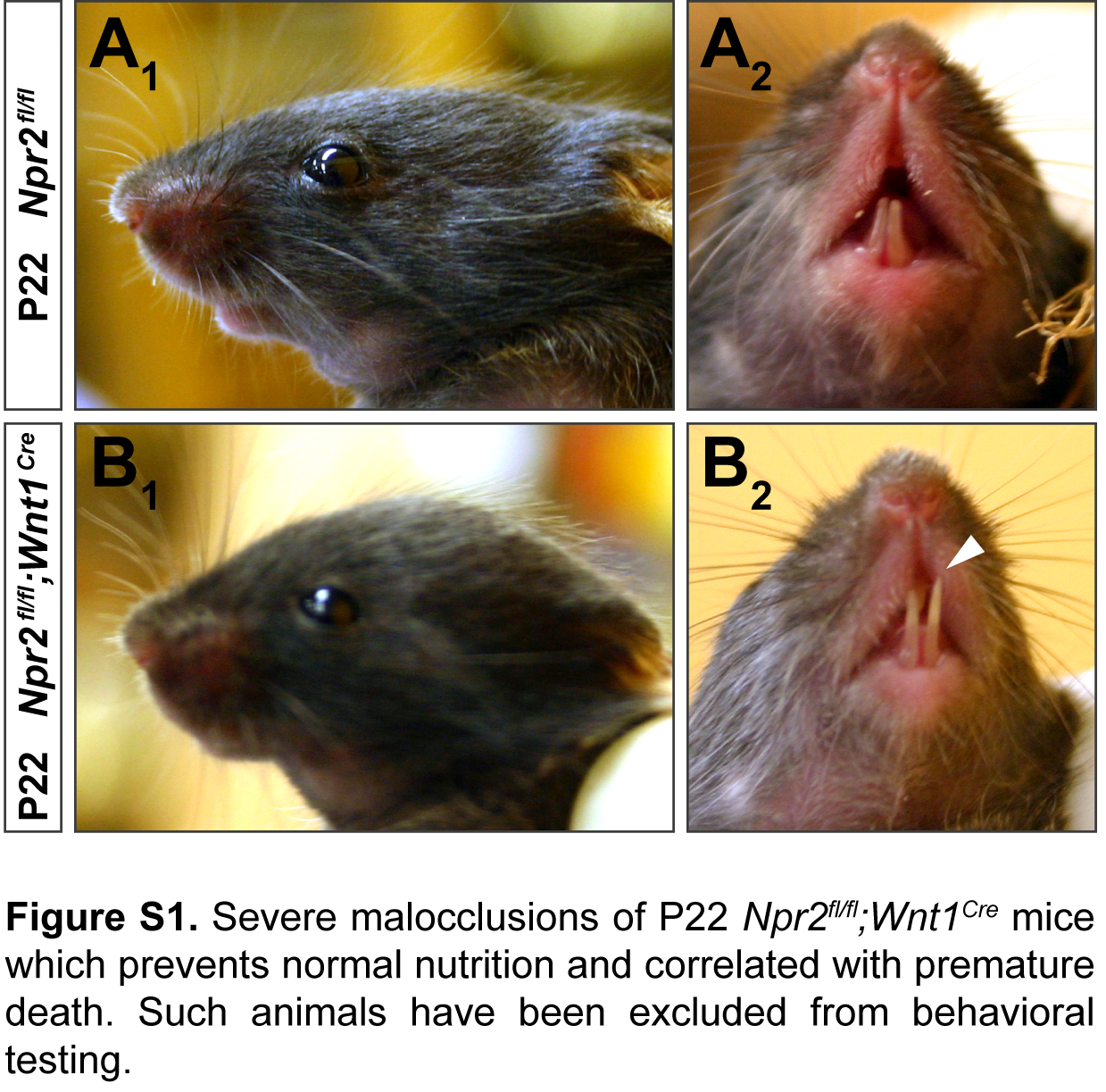

Supplement: Figure S1 — Severe malocclusions of P22 Npr2fl/fl;Wnt1Cre mice which prevents normal nutrition and correlated with premature death. Such animals have been excluded from behavioral testing. [file Image1.tif]

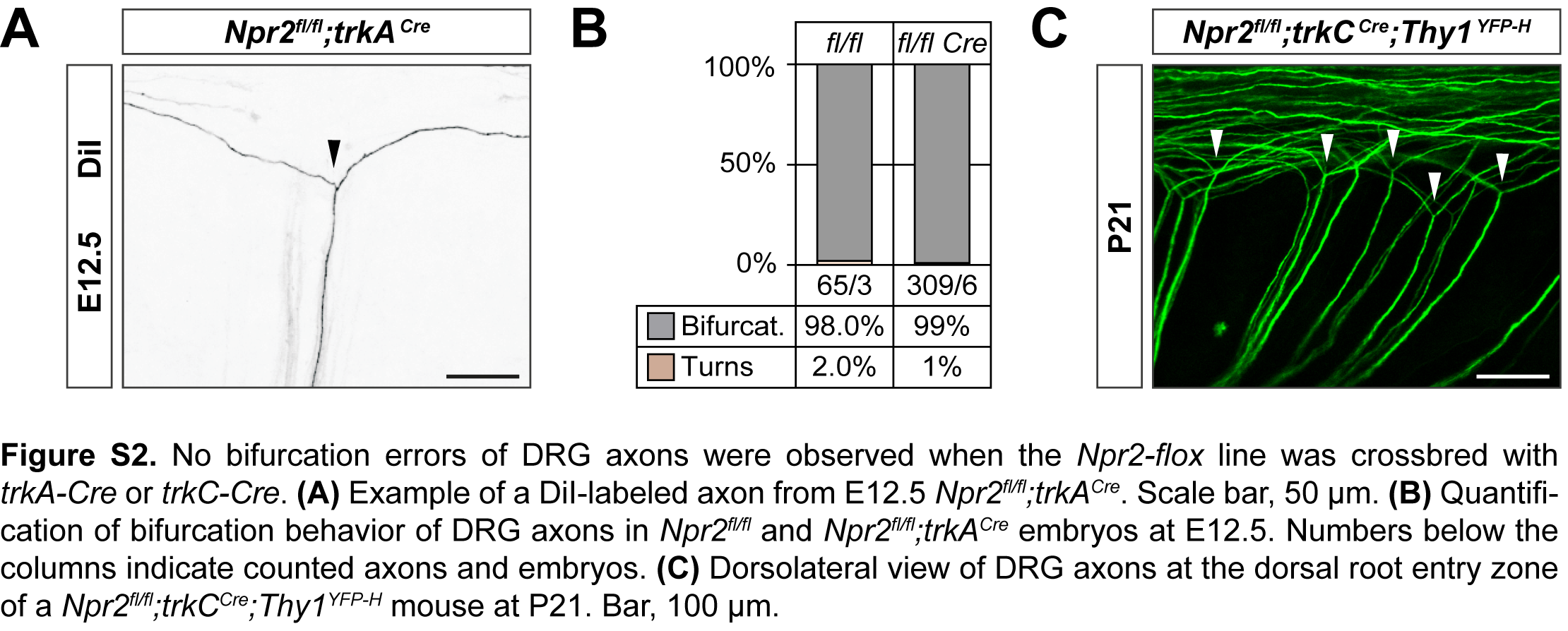

Supplement: Figure S2 — No bifurcation errors of DRG axons were observed when the Npr2-flox line was crossbred with trkA-Cre or trkC-Cre. (A) Example of a DiI-labeled axon from E12.5 Npr2fl/fl;trkACre. Scale bar, 50 μm. (B) Quantification of bifurcation behavior of DRG axons in Npr2fl/fl and Npr2fl/fl;trkACre embryos at E12.5. Numbers below the columns indicate counted axons and embryos. (C) Dorsolateral view of DRG axons at the dorsal root entry zone of a Npr2fl/fl;trkCCre;Thy1YFP−H mouse at P21. Bar, 100 μm. [file Image2.tif]
