# Supplementary material for: The Absence of Sensory Axon Bifurcation Affects Nociception and Termination Fields of Afferents in the Spinal Cord
Source: Front Mol Neurosci. 2018 Feb 8;11:19. doi: 10.3389/fnmol.2018.00019 (PMC5809486; doi:10.3389/fnmol.2018.00019)
Supplement: Table S1 — C-fiber ex vivo skin nerve preparation recorded from the hindpaw hairy skin. C-mechanoheat (CMH), C-mechanoheatcold (CMHC), and C-mechanocold (CMC) fibers were recorded from Npr2fl/fl;Wnt1Cre mice and littermate controls. Mean CVs are shown ± SEM. [file Table1.docx]

**Table S1.** C-fiber *ex vivo* skin nerve preparation recorded from the hindpaw hairy skin. C-mechanoheat (CMH), C-mechanoheatcold (CMHC) and C-mechanocold (CMC) fibers were recorded from *Npr2^fl/fl^;Wnt1^Cre^* mice and littermate controls. Mean CVs are shown +/- SEM.

|  | ***Npr2^fl/fl^*** | | ***Npr2^fl/fl^;Wnt1^Cre^*** | |
| --- | --- | --- | --- | --- |
| **Fiber Type** | **% Total** | **CV m/s** | **% Total** | **CV m/s** |
| **CMH** | 63.5  (21/33) | 0.45 +/-0.06 | 62.5  (15/24) | 0.41 +/-0.05 |
| **CMHC** | 24.5  (8/33) | 0.27 +/-0.03 | 25  (6/24) | 0.49 +/-0.13 |
| **CMC** | 12  (4/33) | 0.78 +/-0.15 | 12.5  (3/24) | 0.38 +/-0.15 |
